# Claudin-7 deficiency promotes stemness properties in colorectal cancer through Sox9-mediated Wnt/β-catenin signalling

**DOI:** 10.1186/s12967-021-02983-3

**Published:** 2021-07-19

**Authors:** Chang Xu, Yu-han Ding, Kun Wang, Mengdi Hao, Huimin Li, Lei Ding

**Affiliations:** 1 Department of Oncology, Beijing Shijitan Hospital, Capital Medical University, Beijing, 100038 China; 2 Department of Hepato-Pancreato-Biliary Surgery, Key Laboratory of Carcinogenesis and Translational Research, Ministry of Education/Beijing , Peking University Cancer Hospital and Institute, Beijing, China

**Keywords:** Claudin-7, Colorectal cancer, Cancer stem cells, Epithelial–mesenchymal transition, Wnt/β-catenin

## Abstract

**Background:**

Colorectal cancer (CRC) is a common malignant tumour of the digestive tract that is characterized by high patient morbidity and mortality rates. Claudin-7 (Cldn7), a tight junction protein, was recently reported to function as a candidate tumour suppressor gene in CRC. Our previous study demonstrated that the large intestine of C57/BL6 mice showed intestinal adenomas and abnormal Ki67 expression and distribution in the intestinal crypt when Cldn7 was knocked out. The aim of this study was to further investigate whether Cldn7 deficiency has non-tight junction functions, affects intestinal stemness properties, promotes CRC and to determine the specific mechanism.

**Methods:**

Cell proliferation assays, migration assays, apoptosis assays, tumour sphere formation assays in vitro*,* and subcutaneous xenograft models in vivo were used to determine the effects of Cldn7 knockdown on the biological characteristics of CRC stem cells. Western blotting, qPCR and immunofluorescence staining were performed to identify the epithelial-mesenchymal transition and the activation of Wnt/β-catenin pathway in CRC stem cells. Cldn7 inducible conditional gene knockout mice and immunohistochemical staining further verified this hypothesis in vivo. The mechanism and target of Cldn7 were determined by performing a chromatin immunoprecipitation (ChIP) assay and coimmunoprecipitation (CoIP) assay.

**Results:**

Cldn7 knock down in CRC stem cells promoted cell proliferation, migration, and globular growth in serum-free medium and the ability to form xenograft tumours; cell apoptosis was inhibited, while the cellular epithelial-mesenchymal transition was also observed. These changes in cell characteristics were achieved by activating the Wnt/β-catenin pathway and promoting the expression of downstream target genes after β-catenin entry into the nucleus, as observed in CRC cell lines and Cldn7 gene knockout mouse experiments. Using ChIP and CoIP experiments, we initially found that Cldn7 and Sox9 interacted at the protein level to activate the Wnt/β-catenin pathway.

**Conclusions:**

Based on our research, Cldn7 deficiency confers stemness properties in CRC through Sox9-mediated Wnt/β-catenin signalling. This result clarifies that Cldn7 plays an inhibitory role in CRC and reveals a possible molecular mechanism, which is conducive to further research on Cldn7 and cancer stem cells.

## Background

Claudin proteins are important components of tight junctions in cells. Their abnormal expression can lead to decreased cell adhesion, structural damage and impaired epithelial and endothelial cell function [1]. Claudin-7 (Cldn7) is a member of the claudin family, and its basic function is to maintain cell polarity and form tight junctions [2]. Low expression of the Cldn7 protein is found in a variety of malignant tumours, such as lung cancer, breast cancer, pancreatic cancer, and colorectal cancer (CRC) [3–6].

Low expression of Cldn7 has significant correlation with CRC or the epithelial-mesenchymal transition (EMT) in CRC [7]. Low Cldn7 expression is also associated with poor differentiation, lymph node metastasis, and distant organ metastasis in CRC [8]. Therefore, Cldn7 is considered by most scholars to play an inhibitory role in colorectal inflammation and CRC [9, 10] and to participate in the occurrence and development of CRC, but the specific mechanism remains unclear.

A study on the Cldn7 protein distribution [11] showed that Cldn7 is different from other claudin proteins that are only located at the top of intestinal epithelial cells; Cldn7 is expressed at high levels in the lateral and basal parts of epithelial cells, especially in the intestinal crypts where the stem cells are dense. Fujita [12] examined the expression of various claudin proteins in the intestines of mice and found that Cldn7 not only appears in the same area as occludin but is also expressed at high levels in the basement membrane of mouse intestinal epithelial cells and intestinal crypts. In addition, Cldn7 has functions other than its role in tight junctions. It can interact with matrix metalloproteinases (MMPs)-3/7 and affect intercellular signal transduction [13]. Due to the particular location and function of Cldn7, it may have a function other than its roles in tight junctions that is related to cancer stem cells (CSCs) in CRC. A small number of CSCs are present in malignant tumour tissues. These cells have the potential for self-renewal, immortalization and multidirectional differentiation and are tumour-initiating cells (TICs) [14, 15]. The current study found that tumour EMT is related to CSCs, and tumours with low expression of claudin proteins produce more immature stem cells (including CSCs) while participating in the EMT [16]. The Wnt/β-catenin/Tcf signalling pathway plays an important role in CSC self-renewal and differentiation and is thought to be involved in the occurrence and development of CRC [17, 18].

In this study, we performed morphological experiments and molecular biology experiments in vivo and in vitro to elucidate the molecular mechanisms by which Cldn7 deficiency confers stem cell characteristics, regulates CSC-related signalling pathways, and ultimately promotes tumourigenesis in CRC.

## Materials and methods

### Cell culture and transfection

Human colon cancer cell lines HCT116, HT29, SW620 and SW480 were purchased from ATCC (USA). HCT116 and SW620 cells were cultured in RPMI 1640 medium (Gibco, USA), HT29 cells and SW480 cells were cultured in DMEM/F12 (Gibco, USA). All cells were cultured in medium supplemented with 10% FBS (Gibco, USA) in humidified air at 37 °C with 5% CO_2_. The comparison of Cldn7 knockdown efficiency between three lentiviral shRNA vectors (sequence #1:5′-ATGGGTGGAGGCATAATTT-3′; sequence #2: 5′-CTAAGTCCAACTCTTCCAA-3′; and sequence #3: 5′-GCTCCTATGCGGGT GACAA-3′) was documented in our previous studies [19]. We selected lentiviral shRNA2 with the highest transfection efficiency and the control vector (sequence: 5′-TTCTCCGAACG TGTCACGT-3′) to transfect HCT116^CD133+CD44+^ cells. The cells were collected after transfection for qRT-PCR and Western blot analysis.

### Flow cytometry

Cell suspensions of HCT116 cells in logarithmic growth phase were collected, washed and centrifuged. PE-labelled anti-CD133 (eBioscience, USA) and FITC-labelled anti-CD44 (Biotech, China) antibodies were then added and incubated for 20 min at room temperature. Cell suspensions without added antibodies were used as a control. The cells were resuspended and subjected to flow cytometry analysis or flow sorting. CD133- and CD44-positive cells were sorted and cultured in complete medium containing penicillin and streptomycin (Gibco, USA).

### Cell proliferation assay

Cell proliferation assays were performed using a cell counting kit (CCK-8) assay (Dojindo Laboratories, Japan). Cldn7 knockdown HCT116^CD133+CD44+^ cells and control cells were seeded in 96-well plates at a density of 2 × 10^3^ cells per well. After 24, 48, 72 and 96 h, CCK-8 reagent (10 μL/well) was added to each well, and the cells were incubated with the reagent for 2 h at 37 °C. The absorbance of the cells at 450 nm was then measured using a microplate reader.

### Cell migration assay

Cldn7 knockdown HCT116^CD133+CD44+^ cells and control cells were seeded evenly into 6-well plates. After the bottom of the well was covered with cells, the cell monolayer was lightly scraped using a 10-μL pipette tip to form a scratch. After washing away the floating cells with PBS, the cells were further cultured in serum-free medium at 37 °C. At 0, 6, 12, and 24 h, scratch wound healing was observed with an inverted microscope and photographed.

### Cell apoptosis assay

Apoptosis assays were performed using a Caspase-Glo® 3/7 kit (Promega, USA). Cldn7 knockdown HCT116^CD133+CD44+^ cells and control cells were seeded in 96-well plates at a density of 10^4^ cells per well. After 24 h of cell culture, Caspase-Glo® Buffer and Caspase-Glo® Substrate were thoroughly mixed at room temperature and then added to each well (100 μL/well). The mixture was shaken gently for 30 s and incubated for 1 h at room temperature. Then, the fluorescence values of the cells were measured with a microplate reader.

### Tumour sphere formation assay

Cldn7 knockdown HCT116^CD133+CD44+^ cells and control cells were seeded in 6-well plates at a density of 10^4^ cells per well. The cells were cultured in DMEM/F12 containing 20 ng/mL EGF (Peprotech, USA), 20 ng/mL basic FGF (Peprotech, USA), 2% B27 (Gibco, USA) and 1% double antibiotics (penicillin and streptomycin, Gibco, USA). The medium was changed every three days. Tumour sphere growth was observed and photographed. Some tumour spheres were inoculated in complete medium containing serum, and the growth of the cells was continuously observed.

### In vivo subcutaneous xenograft model

Six- to eight-week-old male and female BALB/c nude mice weighing 17–20 g were used for this experiment. Cldn7 knockdown HCT116^CD133+CD44+^ cells and control cells were diluted to 1 × 10^6^ cells/ml, mixed evenly and injected into the back of each nude mouse (each injection was 100 μL). Then, the growth of the subcutaneously implanted tumours was recorded. The long and short diameters of the tumours were recorded every 3 days. The tumour volume was calculated as V = 1/2 the long diameter × the short diameter^2^. After 30 days, the nude mice were sacrificed by cervical dislocation, and the tumours were removed and photographed. After the tumour volumes were recorded, the tumour formation rate was calculated, and growth curves for subcutaneous tumour formation were drawn.

### Western blotting

Total protein was extracted from cells and tissues, and the protein concentrations were measured using a BCA kit (Thermo Fisher Scientific, USA). The proteins were then separated by sodium dodecyl sulfate (SDS)-polyacrylamide gel electrophoresis and transferred onto a nitrocellulose (NC) membrane. The membranes were blocked with Tris-buffered saline-Tween (TBST) solution containing 5% skim milk. Next, the membranes were incubated with anti-Cldn7 antibody (ab27487, 1:1000; Abcam), anti-Sox9 antibody (ab185230, Abcam), anti-Olfm4 antibody (ab105861, Abcam), anti-Ki67 antibody (ab16667, Abcam), anti-β-catenin antibody (ab32572, Abcam), anti-cyclin D1 antibody (ab134175, Abcam), anti-C-myc antibody (ab32072, Abcam), anti-E-cadherin antibody (ab40772, Abcam), anti-Snail-1 antibody (ab180714, Abcam) or anti-vimentin antibody (ab8978, Abcam) at 4 °C overnight, followed by an incubation with a donkey anti-rabbit IgG antibody (ab175780; Abcam) or anti-goat IgG antibody (ab175780; Abcam). Finally, a Western blot scanner was used to visualize the blots. Glyceraldehyde-3-phosphate dehydrogenase (GAPDH) was used as the cytoplasmic internal reference, and lamin B1 was used as the nuclear internal reference.

### RNA extraction and quantitative reverse-transcription polymerase chain reaction (qPCR)

Total RNA was extracted from Cldn7 knockdown HCT116^CD133+CD44+^ cells and control cells using TRIzol reagent (Thermo Fisher Scientific, USA). Reverse transcription was performed using SYBR Green PCR Master Mix (TOYOBO, Japan) and an ABI7500 system (Thermo Fisher Scientific, USA). Primers were designed by Sangon Biotech (Shanghai, China). The sequences of the Cldn7 primers were 5′-AAAGTGAAGAAGGCCCGTATA-3′ (forward primer) and 5′-TAATGTTGGTAGGGATCAAAGG-3′ (reverse primer). The Sox9 forward primer was 5′-GCACATCAAGACGGAGCAG-3′, and the reverse primer was 5′-GTAGGTGAAGGTGGAGTAGAGG-3′. The Olfm4 forward primer was 5′-GTAGGTGAAGGTGGAGTAGAGG-3′, and the reverse primer was 5′-GGACGACAGGGGTGTTTTGAT-3′. The Ki67 forward primer was 5′-CACTCCACCTGTCCTGAA-3′, and the reverse primer was 5′-TGTTGACTTCGGCTGATAG-3′. The β-catenin forward primer was 5’-ACACCAAGAAGCAGAGATG-3′, and the reverse primer was 5′-ACGAACAAGCAACTGAACT-3′. The c-Myc forward primer was 5′-ACCGAGGAGAATGTCAAGA-3′, and the reverse primer was 5′-CGCACAAGAGTTCCGTAG-3′. The cyclin D1 forward primer was 5′-TGAACAAGCTCAAGTGGAA-3′, and the reverse primer was 5’-GCGGTAGTAGGACAGGAA-3′. The E-cadherin forward primer was 5′-TACACTGCCCAGGAGCCAGA-3′, and the reverse primer was 5′-TGGCACCAGTGTCCGGATTA-3′. The Snail-1 forward primer was 5′-CCACAAGCACCAAGAGTC-3′, and the reverse primer was 5′-AGAGGACACAGAACCAGAA-3′. The vimentin forward primer was 5′-CTTTGCCGTTGAAGCTGCTA-3′, and the reverse primer was 5′-GAAGGTGACGAGCCATTTCC-3′. The GAPDH forward primer was 5′-TGACTTCAACAGCGACACCCA-3′, and the reverse primer was 5′-CACCCTGTTGCTGTAGCCAAA-3′. After normalization to GAPDH gene expression, the gene expression levels were analysed using the comparative threshold cycle (2^−ΔΔCt^) method.

### Immunofluorescence (IF) staining

Cldn7 knockdown HCT116^CD133+CD44+^cells and control cells were cultured in a 24-well confocal chamber (Nest, China), fixed with 4% paraformaldehyde at room temperature for 20 min and permeabilized with 0.1% Triton X-100 at room temperature for 3 min. The cells were then blocked with 1% BSA for 60 min and incubated with primary antibodies against β-catenin overnight at 4 °C. After washing, the cells were incubated with Alexa Fluor 594-conjugated goat anti-rabbit IgG (1:400, ZSGB-BIO, China) for 2 h at room temperature in the dark. Finally, DAPI was added to each chamber to stain the nuclei, and a fluorescent anti-quenching agent was added. Cellular fluorescence staining was observed using a laser scanning confocal microscope (Nikon, Japan).

### Cldn7 inducible conditional gene knockout mice

We constructed Cldn7-floxed mice and then crossed them with villin-CreERT2 mice to obtain Cldn7 inducible intestinal conditional gene knockout mice (Cldn7 ICKO mice, genotype: Cldn7^fl/fl^; villin-CreERT2). The construction method and identification method were the same as our previous study [20]. Six- to eight-week-old Cldn7 ICKO mice were selected, and each mouse was injected intraperitoneally with 100 μL of tamoxifen (10 mg/mL) every 5 days to activate Cre recombinase expression and achieve Cldn7 gene knockout [20]. Cldn7^fl/fl^; villin-CreW mice, which were the control group, were administered the same dose. The dying mice and the control mice were sacrificed by cervical dislocation. The large intestine and small intestine were harvested, and the intestinal tract was observed using hematoxylin–eosin (HE) and immunohistochemical (IHC) staining.

### IHC staining

All tumour, paracancerous, and metastatic tissues were embedded into wax blocks and cut into paraffin sections. Xylene was used to dewax the paraffin slices. Different concentration gradients of alcohol were used for hydration. Tissue sections were incubated for 10 min in 3% H_2_O_2_ and washed with 0.01 mol/L PBS. The sections were then incubated with anti-Cldn7 antibody, anti-Sox9 antibody, anti-β-catenin antibody, anti-cyclin D1 antibody or anti-c-Myc antibody, followed by incubation with the corresponding secondary antibody (ab175780, Abcam). Afterwards, the proteins were developed in diaminobenzidine (DAB) for colouration. Haematoxylin was used to stain the nuclei, and different concentration gradients of alcohol were used for dehydration. Neutral gum was used to seal the slides.

### ChIP assay

A ChIP assay was performed according to the manufacturer’s instructions (SimpleChIP Plus Sonication Chromatin IP Kit, CST).

An HCT116^CD133+CD44+^ cell pellet was subjected to cross-linking, cross-linking suspension, cell and nuclear lysis, ultrasonic disruption, and chromatin dilution. The lysate was immunoprecipitated with Sox9 antibody (Abcam, USA). Then, the purified DNA was quantitatively pulled down using qPCR. The sequences of the Cldn7 primers were 5’-TGTTGGGAAGAAAGGAAGG-3’ (forward primer) and 5’-CCAGGTGAGGAGGAAGAA-3’ (reverse primer).

### CoIP assay

Total protein was extracted from Cldn7 knockdown HCT116^CD133+CD44+^ cells and control cells. Then, a BCA Protein Assay Kit (Solarbio, Beijing) was used to determine the protein concentrations, and the sample protein concentrations were adjusted to a consistent level. Then, 20 μL of Protein A/G beads were added to 500 μL of protein sample and incubated at 4 °C for 30 min. The supernatants were incubated with anti-Cldn7, anti-Sox9 or anti-IgG antibodies for 1 h at 4 °C. Then, 20 μL of Protein A/G beads were added and incubated overnight at 4 °C. The beads were then washed four times with RIPA buffer, and 50 μL of 1 × loading buffer were added to the final pellet and incubated at 100 °C for 10 min. Bound proteins were eluted from the beads in SDS sample buffer and analysed using Western blotting.

### CRC, paracancerous, metastatic cancer tissue samples

CRC tissue chips HColA150CS02 (Shanghai Outdo Biotech Co.,Ltd., China) and HLinAde075Met01 (Shanghai Outdo Biotech Co.,Ltd., China) were used in this study to analyze the expression of Sox9 in adjacent normal tissues, CRC and metastatic cancer tissues.

The demographic characteristics of these patients are shown in http://www.superchip.com.cn/biology/category_309/1272.html and http://www.superchip.com.cn/biology/category_309/1255.html. CRC tissues were collected from 75 patients with CRC who did not undergo preoperative radiotherapy or chemotherapy. For each patient, adjacent tissues located more than 5 cm away from the tumour were also collected. All of these adjacent samples were confirmed to be normal colorectal tissues by pathology. Remote metastatic CRC tissues were also collected, such as CRC liver metastasis tissues and lung metastasis tissues. IHC staining was used to detect the expression of Sox9 in all samples. Oral informed consent was obtained from all patients. This research was reviewed and approved by the Medical Ethics Committee of the Beijing Shijitan Hospital Affiliated Capital Medical University Institutional Review Board.

### Statistical analyses

Statistical analyses were performed using IBM SPSS version 17.0 and GraphPad Prism version 6.0. All data are presented as the means ± standard deviations. The differences between Cldn7 knockdown HCT116^CD133+CD44+^ cells and control cells were analysed using Student’s t-test. The Sox9 expression positive rate in different tissues was analysed using the X^2^ test. Differences were considered significant when P < 0.05.

## Results

### Construction of CRC stem cells with stable Cldn7 knockdown

**Fig. 1:**
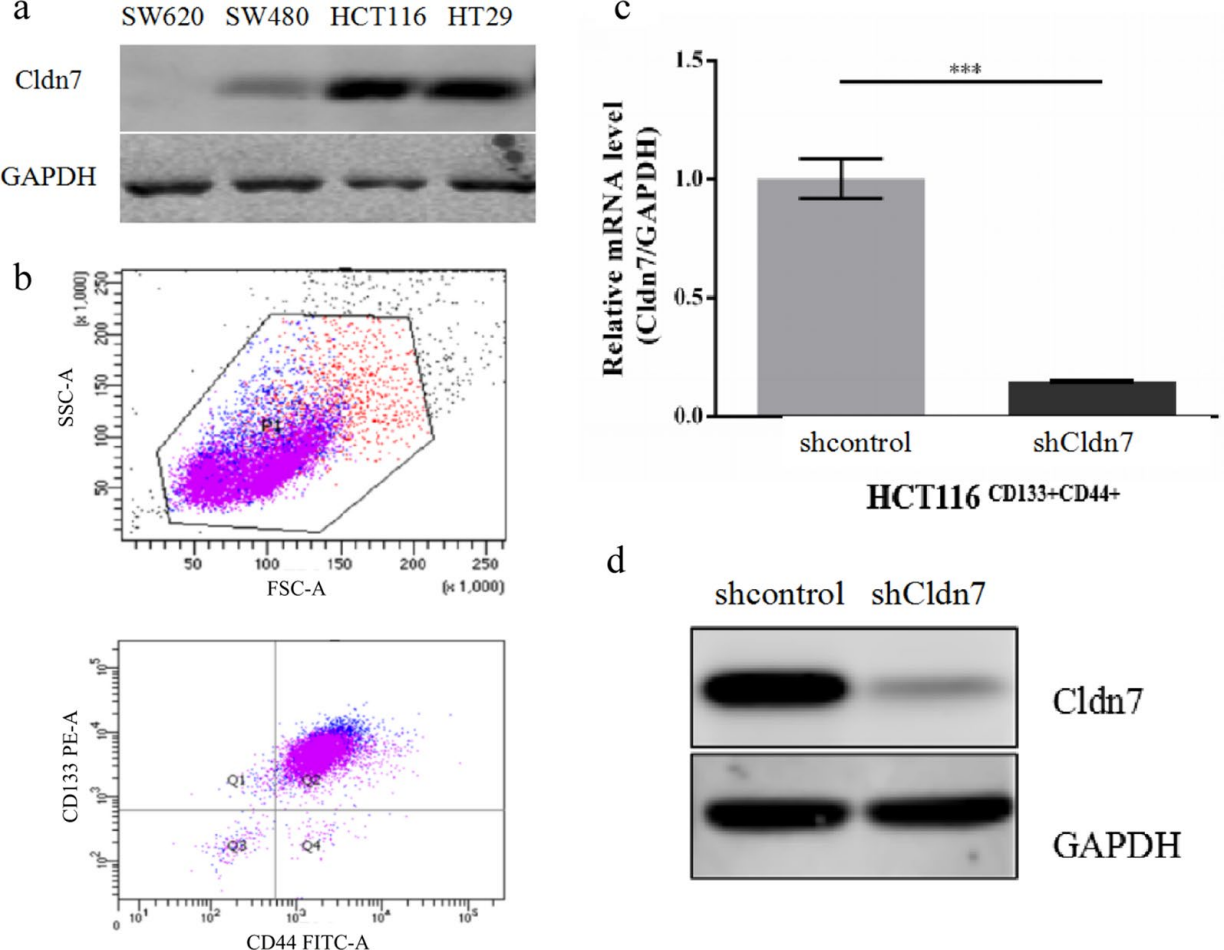
Construction of CRC stem cells with stable Cldn7 knockdown. **a** Cldn7 expression in SW620, SW480, HCT116, and HT29 cell lines. **b** HCT116^CD133+CD44+^ cells were analysed using flow cytometry after stable culture, and the CD133 and CD44 double-positive rate was 97%. **c**, **d** The efficiency of Cldn7 knockdown in HCT116^CD133+CD44+^ cells was detected using qPCR and Western blotting

We identified a group of CRC stem cells based on the coexpression of CD133 and CD44 markers and established stable Cldn7 knockdown stem cells. Cldn7 protein expression was detected in four CRC cell lines (SW620, SW480, HCT116, and HT29) (Fig. 1a). The HCT116 cell line with high Cldn7 expression was selected for further investigation. HCT116^CD133+CD44+^ cells were stably subcultured, and flow cytometry showed that 97% of cells expressed CD133 and CD44 (Fig. 1b). HCT116^CD133+CD44+^ cells were then transfected with lentiviral Cldn7 shRNA2, and lentiviral vectors with nonsilencing shRNA were used as controls. Knockdown efficiency was confirmed by qPCR and Western blotting (Fig. 1c, d), which indicated the successful construction of CRC stem cells with stable Cldn7 knockdown.

### Cldn7 knockdown promoted the tumour-initiating cell (TIC) features and biological behavior of CRC stem cells

**Fig. 2:**
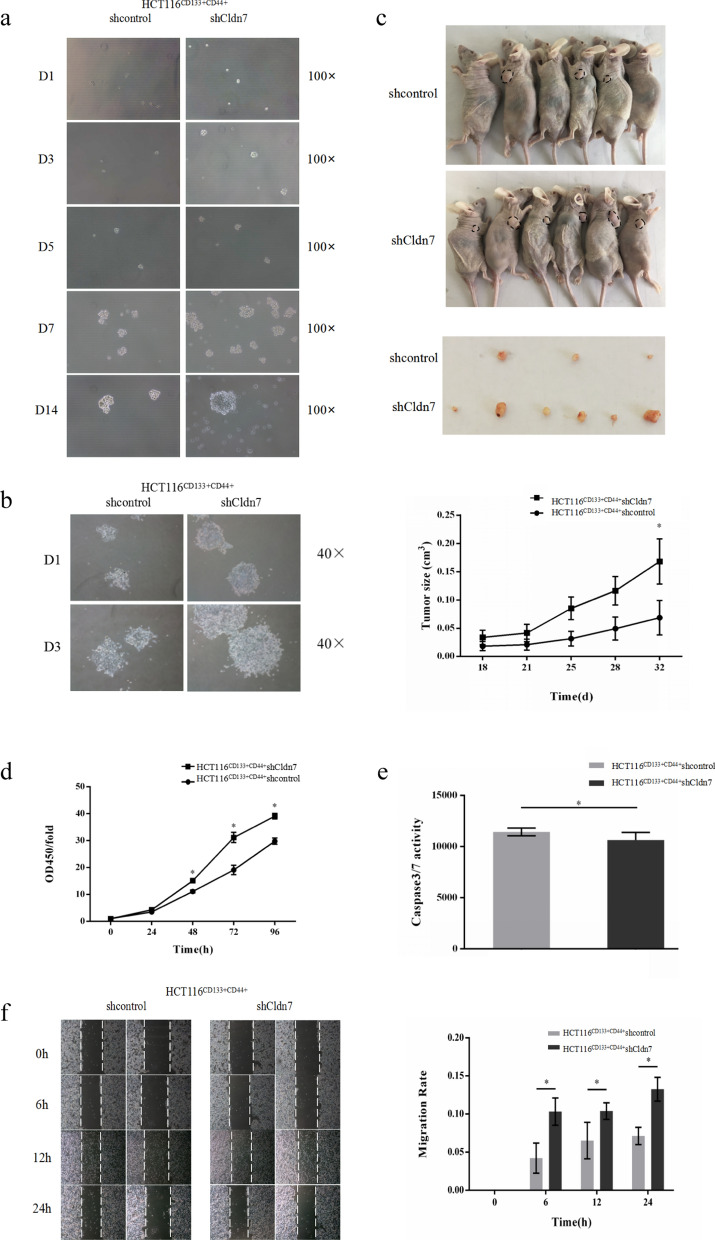
Cldn7 knockdown promoted the TIC features and biological behavior of CRC stem cells. **a** HCT116^CD133+CD44+^ shCldn7 cells formed tumour spheres faster than HCT116^CD133+CD44+^ shcontrol cells. **b** Both cell spheres returned to an adherent state in complete medium, and the cells dissociated from the cell sphere and continued to grow and proliferate. **c** Tumour growth curves showed that the tumour formation rate and tumour volume formed by HCT116^CD133+CD44+^shCldn7 cells were significantly greater than control cells (P < 0.05). **d** Proliferation ability of HCT116^CD133+CD44+^shCldn7 cells was increased (P < 0.05). **e** Apoptosis ability of HCT116^CD133+CD44+^shCldn7 cells was weakened (P < 0.05). **f** Migration ability of HCT116^CD133+CD44+^shCldn7 cells was greater than control cells (P < 0.05)

TICs have the ability to initiate tumour growth and metastasis. We evaluated the ability of spherical growth in vitro and tumour formation in vivo to clarify that HCT116^CD133+CD44+^ cells have TIC properties and analyzed the role of Cldn7 in these properties. According to the non-adherent sphere formation assay, HCT116^CD133+CD44+^shCldn7 cells formed small cell clusters on day 3, and typical neurosphere-like growth was observed on day 5, while small cell masses were maintained for HCT116^CD133+CD44+^shcontrol cells. On day 7 and day 14, both cells were able to form more pronounced cell spheres, but the volume of HCT116^CD133+CD44+^shCldn7 cell spheres was larger than the control group (Fig. 2a). When spheres from the two cell lines were transferred to complete medium containing FBS, both cell spheres returned to the adherent state, and the cells dissociated from the cell sphere and continued to grow and proliferate (Fig. 2b). HCT116^CD133+CD44+^ cells formed tumour spheres in vitro and continued to grow adherently in complete culture medium, indicating that they tolerated serum-free culture conditions, maintained the ability to proliferate and divide and had characteristics of stem cells rather than differentiated cells. Cldn7 knockdown inhibited the stem cell characteristics of HCT116^CD133+CD44+^ cells. CRC stem cells were also inoculated into BALB/c nude mice to form xenograft tumours. As shown in Fig. 2c, compared with HCT116^CD133+CD44+^shcontrol cells, HCT116^CD133+CD44+^shCldn7 cells exhibited a higher tumour formation rate (50% vs. 100%). The tumour volume formed by HCT116^CD133+CD44+^shCldn7 cells was also larger than that formed by control cells (P < 0.05).

We also investigated the effect of Cldn7 expression on CRC stem cell proliferation, apoptosis and migration. At 48, 72 and 96 h, the proliferation rate of HCT116^CD133+CD44+^shCldn7 cells was higher than HCT116^CD133+CD44+^shcontrol cells (P < 0.05) (Fig. 2d). The apoptosis ability of HCT116^CD133+CD44+^shCldn7 cells was reduced (P < 0.05) (Fig. 2e). At 6, 12 and 24 h after scratch wound formation, the mobility based on the rectangular void area between scratches of HCT116^CD133+CD44+^ shCldn7 cells was greater than control cells, suggesting increased migration (P < 0.05) (Fig. 2f).

### Cldn7 knockdown promoted CSC and EMT marker expression in CRC stem cells

**Fig. 3:**
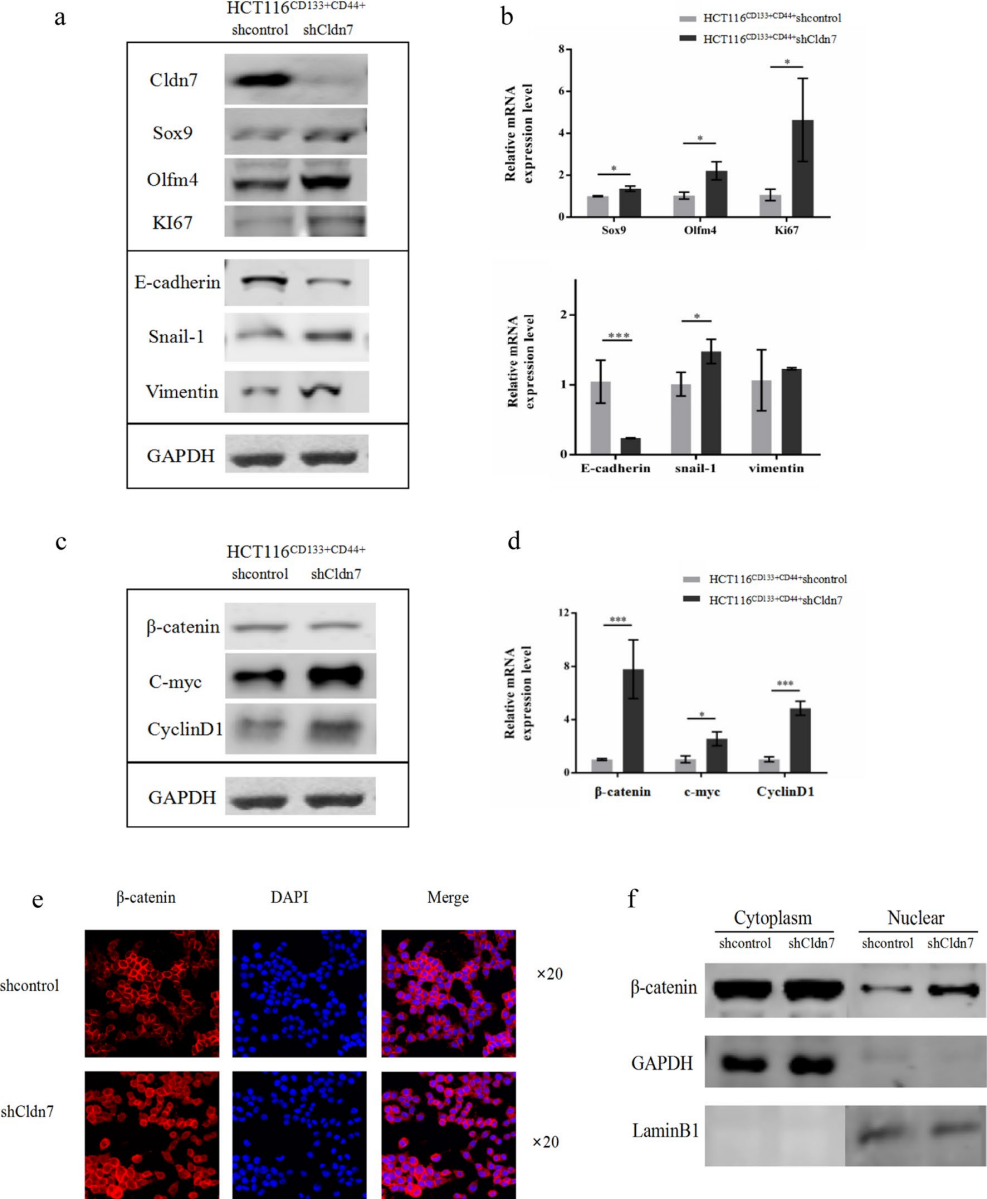
Cldn7 knockdown promoted cancer stem cell marker expression and EMT in CRC stem cells by activating the Wnt/β-catenin pathway. **a**, **b** Sox9, Olfm4 and Ki67 expression increased in HCT116^CD133+CD44+^shCldn7 cells. EMT-related protein E-cadherin was decreased, Snail-1 and vimentin were increased. With the exception of the non obvious increase in vimentin expression, qPCR results were consistent with Western blot results (P < 0.05). **c**, **d** β-catenin protein levels did not change significantly in HCT116^CD133+CD44+^shCldn7 cells, but c-Myc and cyclin D1 expression increased (P < 0.05). The mRNA levels of β-catenin, c-Myc and cyclin D1 in HCT116^CD133+CD44+^shCldn7 cells were all increased (P < 0.05). **e** In HCT116^CD133+CD44+^shCldn7 cells, β-catenin was distributed not only on the cell membrane but also in the cytoplasm and nucleus at low levels. **f** The expression of β-catenin in the cytoplasm of CRC stem cells did not change significantly with the downregulation of Cldn7, but the level of β-catenin in the nucleus increased

With the decrease of Cldn7 expression, expression of CSC markers Sox9, Olfm4 and the proliferation marker Ki67 increased significantly (Fig. 3a). In addition, Cldn7 deficiency downregulated the expression of EMT-related protein E-cadherin and upregulated Snail-1 and vimentin expression (Fig. 3a), suggesting that Cldn7 downregulation promoted the EMT in CRC stem cells. These effects were further supported by qPCR experiments (Fig. 3b). Except for the increase in vimentin expression, the qPCR results were consistent with the Western blot results (P < 0.05).

### Cldn7 knockdown promoted the TIC features of CRC stem cells by activating the Wnt/β-catenin pathway

Compared with control cells, the mRNA levels of β-catenin and its downstream genes c-Myc and cyclin D1 were upregulated in HCT116^CD133+CD44+^shCldn7 cells (Fig. 3d). C-Myc and cyclin D1 protein expression levels were also increased, while the β-catenin levels in HCT116^CD133+CD44+^shCldn7 cells did not change significantly (Fig. 3c).

Because activated β-catenin enters the nucleus to function, we used immunofluorescence experiments and subcellular fractionation experiments to observe whether β-catenin enters the nucleus to induce protein expression. As shown in Fig. 3e, β-catenin was not only distributed on the cell membrane but also accumulated in the cytoplasm and nucleus at low levels. By increasing the protein mass for loading, Western blots did not reveal significant changes in β-catenin levels in the cytoplasm following the downregulation of Cldn7, but β-catenin levels in the nucleus increased (Fig. 3f), which may not result in a significant difference in the total protein level.

### *Cldn7 knockout/knockdown promotes the development of colorectal adenomas and xenograft tumour by activating the Wnt/β-catenin pathway *in vivo

**Fig. 4:**
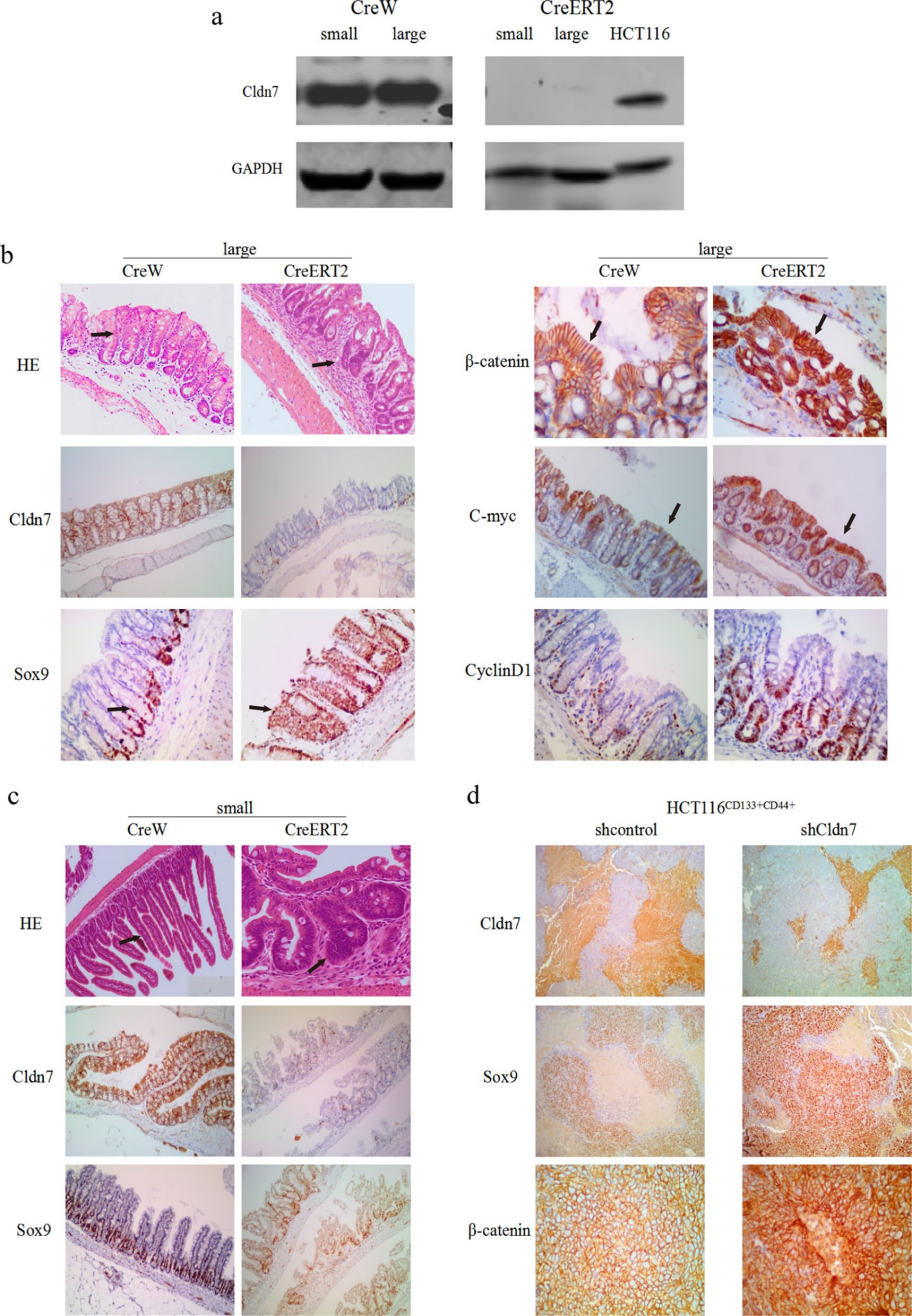
Cldn7 knockout/knockdown promotes the development of colorectal adenomas and xenograft tumours by activating the Wnt/β-catenin pathway in vivo. **a** Cldn7 was knocked out in the small and large intestines of CreERT2 mice. **b**, **c** Extensive and severe inflammatory infiltrates and adenomas were observed in the small and large intestines of CreERT2 mice, while the intestinal tissue of CreW mice was normal (as indicated by the arrow). In the large intestine of CreERT2 mice, Sox9, β-catenin, c-Myc and cyclin D1 expression increased with Cldn7 knockout, and more β-catenin was present in the cytoplasm and nuclei. Activation of the Wnt/β-catenin pathway was not detected in the small intestine of CreERT2 mice, but an increase in Sox9 expression was still observed in the small intestine of CreERT2 mice. **d** In the xenograft tumour tissue formed by HCT116^CD133+CD44+^ shCldn7 cells, Sox9 and β-catenin expression increased, and β-catenin was observed in more cytoplasm and nuclei

Cldn7 ICKO mice (CreERT2 mice) and control mice (CreW mice) were generated and injected with tamoxifen to knock out Cldn7 expression. Western blot showed that Cldn7 in the small and large intestine had been knocked out in CreERT2 mice (HCT116 cells were used as a positive control), while Cldn7 was still expressed in the intestinal tract of CreW mice (Fig. 4a).

In our previous study, CreERT2 mice were apathetic and gradually lost weight after tamoxifen injections, eventually reaching a state of dying. The intestinal crypts of CreERT2 mice were significantly enlarged and dilated, and cells with positive Ki67 expression covered the entire crypt. Proliferating cells were not only confined to the crypt but also distributed throughout the villi [20]. These phenomena were more pronounced in the large intestine, suggesting abnormal intestinal stem cell proliferation, but the mechanism was unclear.

HE staining revealed extensive and severe inflammatory infiltrates and adenomas in the small and large intestines of CreERT2 mice, while the intestinal tissue of CreW mice was normal (Fig. 4b, c). Immunohistochemical staining showed increased levels of Sox9, β-catenin, c-Myc and cyclin D1 in the large intestine of CreERT2 mice, accompanied by changes in the location of their expression. Sox9 was widely distributed along the surface of intestinal villi, c-Myc was clearly distributed in the intestinal crypts and the top of the intestinal villi, and β-catenin was present in the cytoplasm and nuclei, suggesting that cell proliferation was active and extensive. Thus, the balance between cell proliferation and differentiation in the large intestine of Cldn7 CreERT2 mice was disrupted. However, although Sox9 expression increase was observed in the small intestine of CreERT2 mice, activation of the Wnt/β-catenin pathway was not significant, suggesting that Wnt/β-catenin activation mainly occurred in the large intestine. These findings were consistent with previous studies of crypt expansion and Ki67-positive cells mainly appeared in the large intestine (Fig. 4c).

The xenograft tumour tissue in previous study were stained with the same antibodies, showing that Sox9, β-catenin increased, and β-catenin could be observed in more cytoplasm and nuclei of the xenografts formed by HCT116^CD133+CD44+^ shCldn7 (Fig. 4d).

### Negative regulation may present between Cldn7 and Sox9 in CRC

**Fig. 5:**
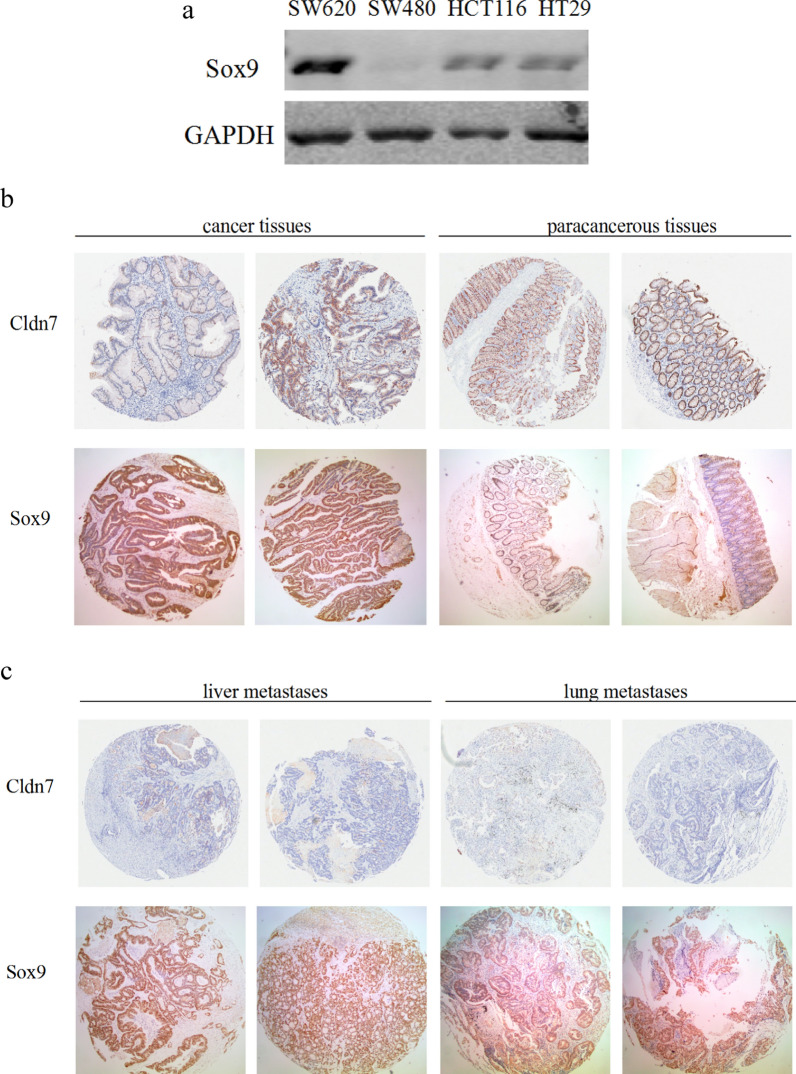
Expression of Cldn7 and Sox9 in CRC cell lines and clinical tissues. **a** Sox9 expression was obvious in SW620 cell lines with low Cldn7 expression, was weak in the high Cldn7-expressing cell lines HCT116 and HT29, and the Sox9 band was virtually undetectable in SW480 cell lines with moderate Cldn7 expression. **b** Cldn7 was expressed at lower levels in CRC tissues than in adjacent tissues, and Sox9 expression was significantly increased in CRC tissues. **c** Cldn7 was expressed at significantly lower levels in metastatic cancer tissues than in primary tumours. Sox9 expression was increased in liver metastasis and lung metastasis tissues

According to the aforementioned experimental results, Sox9 expression was increased with the decrease in Cldn7. This phenomenon was also confirmed in CRC cell lines (Fig. 5a). Sox9 expression was obvious in SW620 cell lines with low Cldn7 expression, while low Sox9 expression was detected in the high Cldn7-expressing cell lines HCT116 and HT29, and very little Sox9 protein was detected in SW480 cell lines with moderate Cldn7 expression.

We used 75 clinical CRC tissues and paracancerous tissues to determine the expression of these two molecules by performing IHC staining. In our previous research, Cldn7 expression was lower in CRC tissues than in adjacent normal tissues [8]. However, Sox9 staining was significantly increased in CRC tissues (Fig. 5b and Table 1). Additionally, in previous study, Cldn7 expression was significantly lower in metastatic cancer tissues than in primary tumours [8], while Sox9 expression was increased in liver metastasis and lung metastasis cancer tissues (Fig. 5c). The positive expression rate in liver metastasis cancer tissues was increased, but the difference was not statistically significant (Table 2). This could be due to Sox9 was already expressed at high levels in primary cancer tissues, and thus the difference was not obvious. Notably, the positive rate of Sox9 expression in lung metastasis tissues was low, and no significant correlation was observed between Cldn7 and Sox9 expression (p > 0.05), potentially because the number of tissues was insufficient.

**Table 1:** Sox9 expression in CRC tissues and paracancerous tissues

Histological type	Number of cases	Number with positive expression	Positive rate	χ^2^	P-value
Paracancerous tissues	75	23	0.31	43.605	0.000*
CRC tissues	75	63	0.84

**Table 2:** Sox9 expression in CRC tissues and metastatic tissues

Histological type	Number of cases	Number with positive expression	Positive rate	χ^2^	P-value
CRC tissues	75	63	0.84		
Liver metastases	18	17	0.94	0.592	0.44
Lung metastases	4	2	0.5	3.011	0.142

### Cldn7 interacts with Sox9 to activate the Wnt/β-catenin pathway

**Fig. 6:**
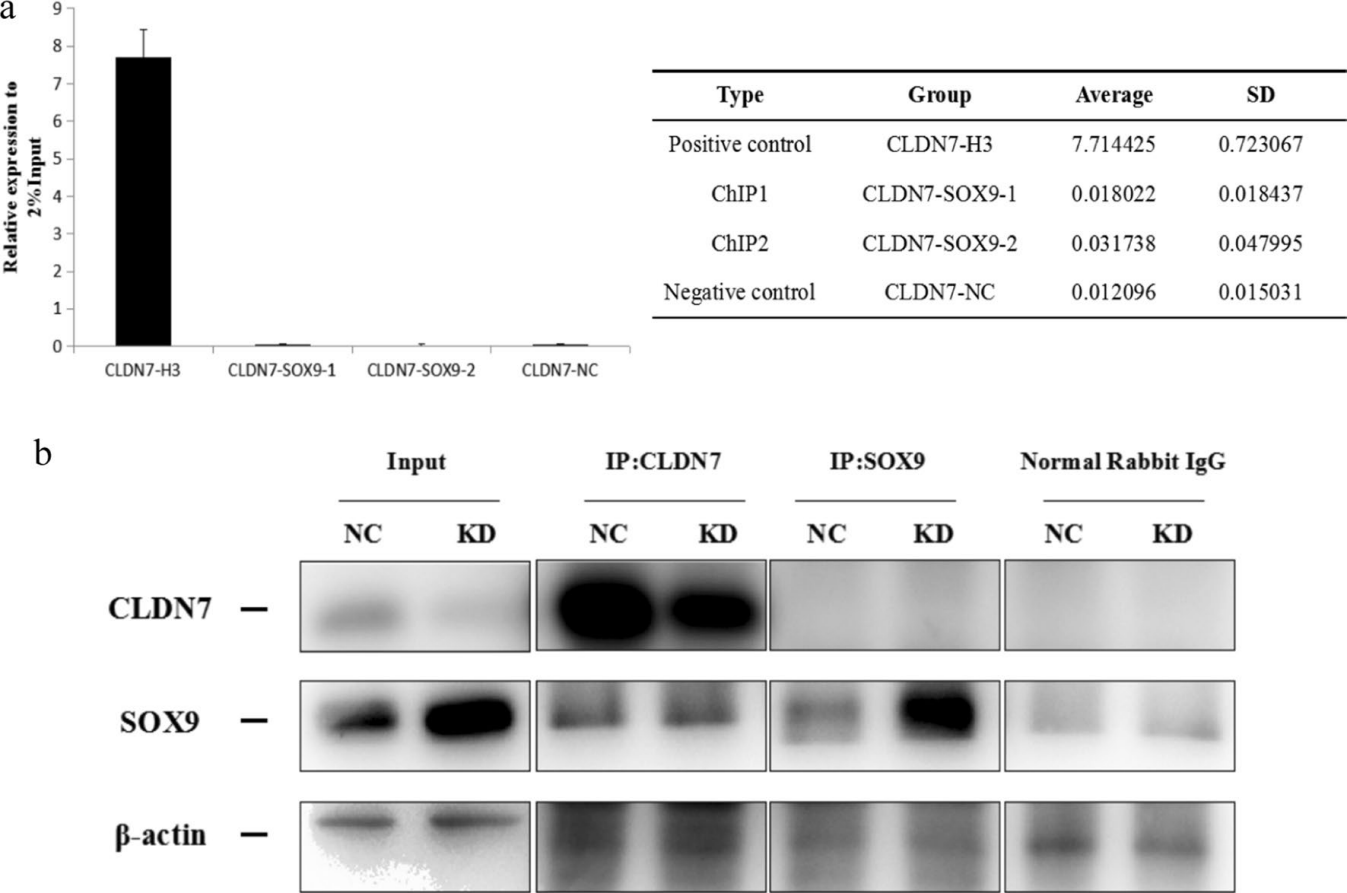
Cldn7 interacts with Sox9 to activate the Wnt/β-catenin pathway. **a** ChIP showed the Sox9 protein failed to bind directly to the Cldn7 promoter region and may be regulated by other means. **b** CoIP results showed Cldn7 and Sox9 interacted at the protein level, but the effect was indirect

Sox9 plays a role in intestinal stem cell proliferation and differentiation in the large intestine, but not the small intestine, and is a transcription factor that regulates the Wnt/β-catenin/Tcf signalling pathway to drive tumourigenesis or the EMT [21–24]. Here, we conducted ChIP and CoIP experiments to determine whether Cldn7 interacts with Sox9 and activates the Wnt/β-catenin pathway. At the transcriptional level, ChIP results are shown in Fig. 6a. The positive control group had a relative expression level of 7.7 to 2% input, and the value of the negative control group was 0.01, which were both within the normal range. The relative expression levels of the Sox9 group were 0.03 and 0.02, which were not significantly different from those of the negative control group. Based on these results, the Sox9 protein failed to bind directly to the Cldn7 promoter region, and the regulation may occur through another mechanism. At the protein level, the CoIP results are shown in Fig. 6b. When using the Cldn7 antibody for IP, both Cldn7 and Sox9 were immunoprecipitated, while the reverse use of Sox9 antibody for IP resulted in the enrichment of only Sox9, but no obvious Cldn7 band, indicating that Cldn7 and Sox9 indirectly interacted at the protein level.

## Discussion

Recent studies have identified a group of CSCs in leukemia, brain tumours, and breast cancer, etc. [25–28], which have the ability to self-renewal and proliferate indefinitely, and these cells are the root cause of tumour initiation, metastasis, and recurrence [29, 30]. In theory, therapy targeting cancer stem cells is more effective than other methods to inhibit tumours and reduce the risk of recurrence and metastasis [31]. Claudin family proteins, the EMT and CSCs constitute an axis that promotes tumourigenesis [32]. Breast cancer with low claudin expression is characterized by differentiation markers deficiency, enrichment of EMT markers, and tumour stem cell-like characteristics [33]. Claudin-low breast tumours show a significant similarity to a "tumourigenic" signature defined using CD44^+^/CD24^−^ breast tumour-initiating stem cell-like cells [34], but the mechanism has not been fully elucidated. In reviewing previous research, no study describing the association between Cldn7 and CRC CSCs was identified, but this topic has great research value.

We previously established inducible intestinal conditional Cldn7 gene knockout mice and surprisingly found intestinal adenomas and enlarged intestinal crypts, as well as increased expression and abnormal distribution of Ki67, suggesting that the balance of intestinal proliferation was disrupted [20]. However, the specific mechanism has not been explored. Therefore, this study was designed to clarify the effect of Cldn7 on the stemness properties of cells in the large intestine, tumour cell proliferation and the specific mechanism that promotes tumourigenesis. In our present research, HCT116^CD133+CD44+^ shcontrol cells and HCT116^CD133+CD44+^ shCldn7 cells can form typical cell spheres in serum-free medium and xenograft tumours in nude mice, indicating that CD133- and CD44-positive CRC cells had obvious stem cell-like phenotypes. Upon Cldn7 knockdown, the formation speed of tumour cell spheres increased, the rate of xenograft tumour formation was higher, cell proliferation and migration were promoted, and cell apoptosis was inhibited, revealing that Cldn7 deficiency promoted the stem cell-like phenotype and functional tumour cell characteristics of CRC stem cells. Cldn7 down regulation also resulted in the enrichment of stem cell markers Sox9, Olfm4 and EMT markers. The final result of EMT is the weakening of intercellular adhesion and the enhancement of invasion, metastasis and stem cell properties [35–37], consistent with the results of our cell-based experiments.

Additionally, we found that Cldn7 low expression activated Wnt/β-catenin pathway, promoted the accumulation of β-catenin in the cytoplasm and nucleus, promoted the transcription and expression of downstream target genes c-Myc and cyclin D1. Cyclin D1 activation accelerates the cell cycle transition from G1 to S phase, thereby promoting cell proliferation [38]; the activation of c-Myc inhibits the apoptotic program [39, 40], which increases cell proliferation and apoptosis resistance. These molecular-level changes were consistent with the results of functional cell-based experiments described above. The Cldn7 ICKO mouse model further confirmed the changes in this pathway in vivo. Activation of the Wnt/β-catenin signalling pathway potentially leads to intestinal epithelial stem cell self-renewal and indefinite proliferation [41–43]. β-catenin destabilization (including activation or point mutations) and β-catenin entry into the nucleus to form complexes with transcription factors such as Tcf4 have been observed in CRC with or without APC deficiency [44, 45]. In addition, Wnt/β-catenin activation may initiate the EMT in CRC [46–48]. These findings supported the hypothesis that Cldn7 promotes stem cell characteristics and the EMT in CRC by activating the Wnt/β-catenin pathway. Interestingly, although adenomas were observed in both the small and large intestine after Cldn7 knockout, Wnt/β-catenin activation and the expression of the downstream target genes cyclin D1 and c-Myc were observed only in the large intestine, indicating different mechanisms of tumourigenesis in the small intestine and large intestine. Activation of the Wnt/β-catenin signalling pathway is the main molecular mechanism underlying CRC tumourigenesis. These results appear to be consistent with other studies showing that Cldn7 knockout leads to hyperproliferation and the EMT in the small intestine, but the expression of Wnt and downstream genes was reduced, and the activation of Wnt/β-catenin signalling rescues the effect of Cldn7 deletion [49]. However, Kim WK et al. proposed that β-catenin was an upstream protein of Cldn7 and that β-catenin activation downregulated the expression of genes related to cell–cell junctions and induced the EMT in CRC [50]. This conclusion also reveals a negative regulatory mechanism between Cldn7 and β-catenin. Cldn7 is also a tumour suppressor in CRC, but the specific regulatory mechanism and up/downstream relationships between the two molecules must be determined in recovery experiments to provide a conservative explanation for these findings.

Sox9 is a transcription factor that regulates the Wnt/β-catenin signalling pathway [51]. The expression of Cldn7 and Sox9 showed an opposite pattern in four colorectal cancer cell lines and the Cldn7 interference cell line. Sox9 expression was upregulated in the small and large intestine of Cldn7 ICKO mice, and clinical pathology also confirmed low Cldn7 expression and high Sox9 expression in CRC tissues. We further analyzed whether Cldn7 interacts with Sox9. The ChIP assay indicated that the Sox9 protein failed to bind directly to the Cldn7 promoter region, and the CoIP results showed that Cldn7 and Sox9 interacted at the protein level. Surprisingly, we did not observe a correlation between Cldn7 and Sox9 expression in clinical specimens, which may be due to the small number of common specimens (n = 28). The study by Darido C et al. revealed the negative regulation between Cldn7 and the β-catenin downstream transcription factor Tcf4 in CRC [52], and Sox9 was identified as a necessary mediator of this effect. This result also agrees with our earlier observations that Cldn7 may activate the Wnt/β-catenin pathway through Sox9. However, in the study by Darido C, Cldn7 overexpression increased CRC cell proliferation and tumourigenesis. Our findings did not confirm this result. This anticancer effect of Cldn7 was fully reflected in clinical tumour tissues, CRC cell lines and Cldn7 inducible conditional gene knockout mouse models. In particular, intestinal adenomas were observed in the large intestine of our Cldn7 gene knockout mice.

This study also has some shortcomings. Cldn7 knockdown enhanced proliferation, migration, and sphere formation but inhibited apoptosis in HCT116^CD133+CD44+^ cells in vitro and promoted tumour formation in vivo. However, the above mentioned phenotypes of SW480 and SW620 cells with low natural Cldn7 expression and HCT116 cells with high Cldn7 expression were not completely opposite and were not completely the same as those of HCT116^CD133+CD44+^ shCldn7 cells. In a study by M Miyo, the proliferation of SW480 cells was reduced compared with HCT116 cells, but the migration capacity was increased [53]. In another study, the proliferation of SW620 and SW480 cells was lower than that of HCT116 and HT29 cells, but the migration and invasion abilities were increased compared with HCT116 and HT29 cells [54]. These findings indicated that different tumour cell lines may have different proliferation and tumourigenesis mechanisms, which may partially be explained by the enrichment and loss of biological phenotypes in the process of establishing different cell lines and might also be related to the different donor sources. Therefore, Cldn7 deficiency promotes CSC characteristics may only be applicable in the HCT116 cell line; its effect on other CRC cell lines should be further explored.

## Conclusion

Cldn7 deficiency enhanced stemness properties in CRC, and the molecular mechanism is likely Sox9-mediated activation of the Wnt/β-catenin signalling pathway. These findings have important implications for the understanding of the non-tight junction function of Clnd7 and provide insights into the roles of tight junction proteins in tumour stem cells. In addition, Cldn7 may be a potential therapeutic target for stem cell therapy of CRC in the future.

## Data Availability

Not applicable.

## Abbreviations

CRC: Colorectal cancer
Cldn7: Claudin-7
EMT: Epithelial-mesenchymal transition
MMP: Matrix metalloproteinase
CSC: Cancer stem cell
TIC: Tumour-initiating cell
ICKO: Inducible conditional knockout
HE: Hematoxylin–eosin
IHC: Immunohistochemical
ChIP: Chromatin immunoprecipitation
CoIP: Coimmunoprecipitation

## References

[1] González-Mariscal L, Betanzos A, Nava P, Jaramillo BE (2003). Tight junction proteins. Prog Biophys Mol Biol.

[2] Günzel D, Yu AS (2013). Claudins and the modulation of tight junction permeability. Physiol Rev.

[3] Lu Z, Liu Y, Xu J, Yin HP, Yuan HY, Gu JJ (2018). Immunohistochemical quantification of expression of a tight junction protein, claudin-7, in human lung cancer samples using digital image analysis method. Comput Methods Programs Biomed.

[4] Bernardi MA, Logullo AF, Pasini FS, Nonogaki S, Blumke C, Soares FA (2012). Prognostic significance of CD24 and claudin-7 immunoexpression in ductal invasive breast cancer. Oncol Rep.

[5] Alikanoglu AS, Gunduz S, Demirpence O, Suren D, Gunduz UR, Sezer C (2015). Expression pattern and prognostic significance of claudin 1, 4 and 7 in pancreatic cancer. Asian Pac J Cancer Prev.

[6] Hahn-Strömberg V, Askari S, Ahmad A, Befekadu R, Nilsson TK (2017). Expression of claudin 1, claudin 4, and claudin 7 in colorectal cancer and its relation with CLDN DNA methylation patterns. Tum Biol.

[7] Wang K, Li TY, Xu C, Ding YH, Li WJ, Ding L (2019). Claudin-7 downregulation induces metastasis and invasion in colorectal cancer via the promotion of epithelial-mesenchymal transition. Biochem Biophys Res Commun.

[8] Xu C, Wang XN, Li WJ, Wang K, Ding L (2018). Expression and Clinical Significance of Claudin-7 in Patients With Colorectal Cancer. Technol Cancer Res Treat.

[9] Ding L, Wang L, Sui L, Zhao H, Xu X, Li T (2016). Claudin-7 indirectly regulates the integrin/FAK signaling pathway in human colon cancer tissue. J Hum Genet.

[10] Oshima T, Miwa H, Joh T (2008). Changes in the expression of claudins in active ulcerative colitis. J Gastroenterol Hepatol.

[11] Gonzalez-Mariscal L, Namorado Mdel C, Martin D, Sierra G, Reyes JL (2006). The tight junction proteins claudin-7 and -8 display a different subcellular localization at Henle’s loops and collecting ducts of rabbit kidney. Nephrol Dial Transplant.

[12] Fujita H, Chiba H, Yokozaki H, Sakai N, Sugimoto K, Wada T (2006). Differential expression and subcellular localization of claudin-7, -8, -12, -13, and -15 along the mouse intestine. J Histochem Cytochem.

[13] Ding L, Lu Z, Foreman O, Tatum R, Lu Q, Renegar R (2012). Inflammation and disruption of the mucosal architecture in claudin-7-deficient mice. Gastroenterology.

[14] Reya T, Morrison SJ, Clarke MF, Weissman IL (2001). Stem cells, cancer, and cancer stem cells. Nature.

[15] Thuma F, Zöller M (2013). EpCAM-associated Claudin-7 supports lymphatic spread and drug resistance in rat pancreatic cancer. Int J Cancer.

[16] Marcucci F, Ghezzi P, Rumio C (2017). The role of autophagy in the cross-talk between epithelial-mesenchymal transitioned tumour cells and cancer stem-like cells. Mol Cancer.

[17] Michael B (2013). Crosstalk between Wnt Signaling and RNA Processing in Colorectal Cancer. J Cancer.

[18] Zhu J, Jiang Y, Yang X, Wang S, Xie C, Li X (2017). Wnt/β-catenin pathway mediates (-)-Epigallocatechin-3-gallate (EGCG) inhibition of lung cancer stem cells. BBRC.

[19] Li W, Xu C, Wang K, Ding Y, Ding L (2019). Non-tight junction-related function of claudin-7 in interacting with integrinβ1 to suppress colorectal cancer cell proliferation and migration. Cancer Manag Res.

[20] Chang Xu, Wang K, Ding Y-H, Li W-J, Ding L (2019). Claudin-7 gene knockout causes destruction of intestinal structure and animal death in mice. World J Gastroenterol.

[21] Kimura MS, Mutoh H, Sugano K (2011). SOX9 is expressed in normal stomach, intestinal metaplasia, and gastric carcinoma in humans. J Gastroenterol.

[22] Ma F, Ye H, He HH, Gerrin SJ, Chen S, Tanenbaum BA (2016). SOX9 drives WNT pathway activation in prostate cancer. J Clin Investig.

[23] Liu H, Liu Z, Jiang B, Peng R, Ma Z, Lu J (2015). SOX9 overexpression promotes glioma metastasis via Wnt/β-catenin signaling. Cell Biochem Biophys.

[24] Montorsi L, Guizzetti F, Alecci C, Caporali A, Martello A, Atene CG (2016). Loss of zfp36 expression in colorectal cancer correlates to wnt/ß-catenin activity and enhances epithelial-to-mesenchymal transition through upregulation of ZEB1, SOX9 and MACC1. Oncotarget.

[25] Lapidot T, Sirard C, Vormoor J, Murdoch B, Hoang T, Caceres-Cortes J (1994). A cell initiating human acute myeloid leukaemia after transplantation into SCID mice. Nature.

[26] Bonnet D, Dick JE (1997). Human acute myeloid leukemia is organized as a hierarchy that originates from a primitive hematopoietic cell. Nat Med.

[27] Singh SK, Hawkins C, Clarke ID (2004). Identification of human brain tumour initiating cells. Nature.

[28] Al-Hajj M, Wicha MS, Benito-Hernandez A, Morrison SJ, Clarke MF (2003). Prospective identification of tumourigenic breast cancer cells. Proc Natl Acad Sci USA.

[29] Pardal R, Clarke MF, Morrison SJ (2003). Applying the principles of stem-cell biology to cancer. Nat Rev Cancer.

[30] Clarke MF (2005). Self-renewal and solid tumour stem cells. Biol Blood Marrow Transplant.

[31] Jordan CT, Guzman ML, Noble M (2006). Cancer stem cells. N Engl J Med.

[32] Turksen K (2011). Claudins and Cancer Stem Cells. Stem Cell Reviews and Reports.

[33] Prat A, Parker JS, Karginova O, Fan C, Livasy C, Herschkowitz JI (2010). Phenotypic and molecular characterization of the claudin-low intrinsic subtype of breast cancer. Breast Cancer Res.

[34] Hennessy BT, Gonzalez-Angulo AM, Stemke-Hale K, Gilcrease MZ, Krishnamurthy S, Lee JS (2009). Characterization of a naturally occurring breast cancer subset enriched in epithelial-to-mesenchymal transition and stem cell characteristics. Can Res.

[35] Yang J, Weinberg RA (2008). Epithelial-mesenchymal transition: at the crossroads of development and tumour metastasis. Dev Cell.

[36] Gupta GP, Massagué J (2006). Cancer metastasis: building a framework. Cell.

[37] Acloque H, Adams MS, Fishwick K, Bronnerfraser M, Nieto MA (2009). Epithelial–mesenchymal transitions: the importance of changing cell state in development and disease. Cell..

[38] Tung JN, Chiang CC, Tsai YY, Chou YY, Yeh KT, Lee H (2010). CyclinD1 protein expressed in pterygia is associated with β-catenin protein localization. Mol Vis.

[39] Wang Q, Zhou Y, Rychahou P, Harris JW, Zaytseva YY, Liu J (2018). Deptor is a novel target of Wnt/β-catenin/c-Myc and contributes to colorectal cancer cell growth. Can Res.

[40] Qiao L, Liu X, Tang Y, Zhao Z, Zhang J, Liu H (2018). Knockdown of long non-coding RNA prostate cancer-associated ncRNA transcript 1 inhibits multidrug resistance and c-Myc-dependent aggressiveness in colorectal cancer Caco-2 and HT-29 cells. Mol Cell Biochem.

[41] Losick VP, Morris LX, Fox DT, Spradling A (2011). Drosophila stem cell niches: a decade of discovery suggests a unified view of stem cell regulation. Dev Cell.

[42] Sato T, Van JH, Snippert HJ, Stange DE, Vries RG, Born M (2010). Paneth cells constitute the niche for Lgr5 stem cells in intestinal crypts. Nature.

[43] Clevers H, Nusse R (2012). Wnt/β-Catenin Signaling and Disease. Cell.

[44] Korinek V, Barker N, Morin PJ, Wichen D, Weger R, Kinzler KW (1997). Constitutive transcriptional activation by a beta-catenin-Tcf complex in APC-/- colon carcinoma. Science.

[45] Morin PJ, Sparks AB, Korinek V, Barker N, Clevers H, Vogelstein B (1997). Activation of beta-catenin-Tcf signaling in colon cancer by mutations in beta-catenin or APC. Science.

[46] Sun J, Zhang T, Cheng M, Hong L, Zhang C, Xie M (2019). TRIM29 facilitates the epithelial-to-mesenchymal transition and the progression of colorectal cancer via the activation of the Wnt/β-catenin signaling pathway. J Exp Clin Cancer Res.

[47] Liang G, Fang X, Yang Y, Song Y (2018). Silencing of CEMIP suppresses Wnt/β-catenin/Snail signaling transduction and inhibits EMT program of colorectal cancer cells. Acta Histochem.

[48] Guo YH, Wang LQ, Li B, Xu H, Yang JH, Zheng LS (2016). Wnt/β-catenin pathway transactivates microRNA-150 that promotes EMT of colorectal cancer cells by suppressing CREB signaling. Oncotarget.

[49] Xing T, Benderman LJ, Sabu S, Parker J, Yang J, Lu Q (2020). Tight junction protein claudin-7 is essential for intestinal epithelial stem cell self-renewal and differentiation. Cell Mol Gastroenterol Hepatol.

[50] Kim WK, Kwon Y, Jang M, Park M, Kim J, Cho S (2019). β-catenin activation down-regulates cell-cell junction-related genes and induces epithelial-to-mesenchymal transition in colorectal cancers. Sci Rep.

[51] Bastide P, Darido C, Pannequin J, Kist R, Robine S, Marty-Double C (2007). Sox9 regulates cell proliferation and is required for Paneth cell differentiation in the intestinal epithelium. Cell Biol.

[52] Darido C, Buchert M, Pannequin J, Bastide P, Zalzali H, Mantamadiotis T (2008). Defective Claudin-7 regulation by Tcf-4 and Sox-9 disrupts the polarity and increases the tumourigenicity of colorectal cancer cells. Cancer Res.

[53] Miyo M, Yamamoto H, Konno M, Colvin H, Nishida N, Koseki J (2015). Tumour-suppressive function of SIRT4 in human colorectal cancer. Br J Cancer.

[54] Kang DH, Woo J, Kim H, Kim SY, Ji S, Jaygal G (2020). Prognostic relevance of HJURP expression in patients with surgically resected colorectal cancer. Int J Mol Sci.

